# Supplementary material for: Secretome Analysis of High- and Low-Virulent Bovine Pasteurella multocida Cultured in Different Media
Source: Animals (Basel). 2023 Nov 28;13(23):3683. doi: 10.3390/ani13233683 (PMC10705078; doi:10.3390/ani13233683)
Supplement: Supplementary file 1 [file animals-13-03683-s001.zip › Statement.pdf]

In order to facilitate data analysis, the naming methods of each gene in the proteomics data were consistent with those of the earlier transcriptomics, all of which began with CQ2GL00. The named genome has not been uploaded yet. If you need to know more information about genes, you can consult the base sequence of related genes through the file CQ2-genome, and then find the relevant number in the file CQ2-NCBI. You can enter the number on the website NCBI to obtain information about related genes.

File “Attachment 1_Protein identification list” shows the secretion of each protein detected in this proteomics. For more information on proteomics, please refer to the link in “Data Availability Statement” of the manuscript.

File “pmcq2-vs-pmcq2_m.genediffexpfilter” is a summary table of the differential expression of genes of *Pasteurella multocida* (PmCQ2) in mice in vitro and vivo, which is also the supplementary data of this experiment.

Note:The Tuf protein mentioned in this manuscript is numbered CQ2GL001603 in proteomics and transcriptomics.
