# Supplementary material for: Secretome Analysis of High- and Low-Virulent Bovine Pasteurella multocida Cultured in Different Media
Source: Animals (Basel). 2023 Nov 28;13(23):3683. doi: 10.3390/ani13233683 (PMC10705078; doi:10.3390/ani13233683)
Supplement: Supplementary file 1 [file animals-13-03683-s001.zip › Supplementary tables.pdf]

Supplementary Table S1: The ingredients of BHI and Martin

Ingredient	BHI broths	Martin broths
Proteose Peptone	√	√
Glucose/ Dextrose	√	√
Sodium chloride	√	×
Disodium Phosphate	√	×
Calf Brains Infusion	√	×
Beef Heart Infusion	√	×
Beef Extract	×	√
Yeast Extract	×	√
Sodium Acetate	×	√

Note: "√" means have, "×" means do not have.

Supplementary Table S2: Differential expression proteins of high and low virulence strains

Protein	Description	Fold change (CQ2-M/CQ6-M)	Fold change (CQ2-B/CQ6-B)
CQ2GL000092	hypothetical protein	ND	Down
CQ2GL000697	cyclophilin B	ND	Down
CQ2GL001658	30S ribosomal protein S11	ND	Down
CQ2GL000696	cyclophilin B	Down	Down
CQ2GL000846	fadL	ND	Down
CQ2GL001440	RlpA	Down	Down
CQ2GL000736	pal	ND	Down
CQ2GL000999	DUF882 domainDowncontaining protein	Down	Down
CQ2GL001673	50S ribosomal protein L16	ND	Down
CQ2GL000177	OmpA	1.5366	1.6763
CQ2GL000324	penicillinDownbinding protein activator, LpoA	1.5193	2.0231
CQ2GL000185	sugar ABC transporter substrateDownbinding protein	4.0450	1.9095

ND:Proteins not detected in group CQ2DownM/CQ6DownM

Down:Proteins only identified in group CQ6DownM or CQ6DownB

Supplementary Table S3: Differentially expressed proteins in different media

Protein	Description	Fold change (CQ2-M/CQ2-B)	Fold change (CQ6-M/CQ6-B)
CQ2GL000092	hypothetical protein	ND	Down
CQ2GL000697	cyclophilin B	ND	Down
CQ2GL001414	hypothetical protein	Down	Down
CQ2GL001642	pirin family protein	Down	Down
CQ2GL001658	30S ribosomal protein S11	ND	Down
CQ2GL001677	50S ribosomal protein L2	ND	Down
CQ2GL000187	UGDH	Down	ND
CQ2GL000270	phosphate acetyltransferase	Down	Down
CQ2GL000737	TolB	Down	Down
CQ2GL000846	fadL	ND	Down
CQ2GL002029	fkpA	Down	Down
CQ2GL002076	SABP	Down	Down
CQ2GL000736	pal	ND	Down
CQ2GL000898	TadD	Down	Down
CQ2GL000495	C4Downdicarboxylate ABC transporter substrateDownbinding protein	Down	Down
CQ2GL000589	tbpA	Down	Down
CQ2GL001655	50S ribosomal protein L17	Down	Down
CQ2GL001673	50S ribosomal protein L16	ND	Down
CQ2GL002041	50S ribosomal protein L1	0.2189	0.5784
CQ2GL001815	phosphoenolpyruvate carboxykinase	0.3453	0.4454
CQ2GL000177	OmpA	0.3511	0.4097
CQ2GL000321	OsmY	0.3677	0.6314
CQ2GL000577	porin	0.4870	0.4471
CQ2GL000380	ABC transporter substrateDownbinding protein	0.5001	0.2438
CQ2GL002051	DNADownbinding protein HU, HupA	0.6231	0.5724
CQ2GL002027	glutathione amideDowndependent peroxidase	0.6341	0.6073

ND:Proteins not detected in group CQ2DownM/CQ2DownB or CQ6DownM/CQ6DownB

Down: Proteins only identified in group CQ2DownB or CQ6DownB

Supplementary Table S4: Putative protective antigens

Protein ID	Description	Secretory pathway	Potential protective antigen
CQ2GL000234	DnaK	NonDownclassical secretory pathway	*Francisella tularensis* *Fish nocardiosis/E.coli*
CQ2GL002029	FkpA	Sec/cleaved by SPI	*Neisseria meningitidis* *Glaesserella parasuis*
CQ2GL000675	GAPDH	NonDownclassical secretory pathway	*Edwardsiella tarda* *Streptococcus equi*
CQ2GL001658	30S ribosomal protein S11	NonDownclassical secretory pathway	*Mycoplasma ovipneumoniae*
CQ2GL000236	htrA	Sec/cleaved by SPI	*Haemophilus influenzae*
CQ2GL000424	mdh	Sec/cleaved by SPI	*Brucella spp*
CQ2GL000737	TolB	Sec/cleaved by SPI	*Vibrio alginolyticus*
CQ2GL000177	OmpA	Sec/cleaved by SPI	*Pasteurella multocida*
CQ2GL000577	OmpH	Sec/cleaved by SPI	*Pasteurella multocida*
CQ2GL000770	MipA	Sec/cleaved by SPI	*Enterotoxigenic E. coli*
CQ2GL000736	peptidoglycanDownassociated lipoprotein,pal	Sec/cleaved by SPII	*Legionella pneumophila*
CQ2GL000185	sugar ABC transporter substrateDownbinding protein	Sec/cleaved by SPII	*Bovine brucellosis*
CQ2GL000589	thiamine ABC transporter substrate binding subunit	Tat secretory pathway	*Brucella abortus*
CQ2GL001505	fructoseDownbisphosphate aldolase, FBA	moonlighting	*Candida albicans* *Streptococcus pneumoniae*
CQ2GL000317	pyruvate kinase,PK	moonlighting	*Bifidobacterium longum* *Mycoplasma gallisepticum* *Candida*
CQ2GL001603	elongation factor Tu,EFDownTu	moonlighting	*Streptococcus agalactiae* *Borrelia burgdorferi*
